# Supervising across professions: insights into the art of interprofessional clinical supervision

**DOI:** 10.1186/s12909-026-10355-y

**Published:** 2026-09-09

**Authors:** Lana Zelić, Klara Bolander Laksov, Anders Sondén

**Affiliations:** 1 Department of Clinical Science and Education, Surgical Section, Karolinska Institutet, Sjukhusbacken 10, Södersjukhuset, Stockholm, 118 83 Sweden; 2 Department of Education, Stockholm University, Stockholm, Sweden; 3 Swedish Red Cross University, Stockholm, Sweden; 4 Centre for Engineering Education, Lund University, Lund, Sweden

**Keywords:** Interprofessional supervision (IPS), Interprofessional education (IPE), Interprofessional learning (IPL), Interprofessional training ward (IPTW), Interprofessional collaboration (IPC)

## Abstract

**Background:**

Implementing interprofessional education in clinical settings is a complex but essential undertaking for preparing health professionals for collaborative practice. In interprofessional training wards, students from multiple professions learn with, from, and about each other while providing patient care together. Supervisors are pivotal to this process, yet supervising across professional boundaries introduces challenges that remain underexplored. This study explored how supervisors from different health professions conceptualise and enact interprofessional supervision in interprofessional training wards.

**Methods:**

This qualitative exploratory study involved interviews with 16 supervisors (nurses, physicians, physiotherapists and occupational therapists) from four interprofessional training wards in Sweden. Purposeful sampling ensured a diverse sample. The interviews were transcribed verbatim and analysed using reflexive thematic analysis.

**Results:**

Three themes were developed: *learning to swim in uncharted waters*, *navigating tensions between profession-specific and interprofessional supervision*, and “*just call the nurse”: the unchosen responsibility and unbalanced leadership of nurses*. Across professions, supervisors reported entering interprofessional supervision with little preparation and relying on learning by doing. They described continuously negotiating their supervisory roles while balancing profession-specific expertise with interprofessional goals, relying on improvisation in practice. Nurses’ continuous presence positioned them as central coordinators of student teams, but this role was demanding and under-recognised. Collectively, the findings highlight interprofessional supervision as improvised, negotiated, and unevenly distributed.

**Conclusion:**

Interprofessional supervision is a distinct but under-supported pedagogical practice embedded in everyday clinical work and shaped by individual, organisational, and structural factors. Without clear frameworks and institutional support, supervisory responsibilities risk being unevenly shouldered. Our findings underscore the need to move interprofessional supervision from tacit improvisation to intentional, shared practice. Investing in supervisor preparation, shared pedagogical frameworks and organisational support is essential for sustaining high-quality interprofessional learning in clinical training wards.

**Trial registration:**

Not applicable. This is a qualitative study and not a clinical trial.

**Supplementary Information:**

The online version contains supplementary material available at https://doi.org/10.1186/s12909-026-10355-y.

## Background

The World Health Organization has identified poor collaboration among health professionals as a major contributor to adverse events and emphasised the importance of fostering strong interprofessional working relationships [9]. In response, interprofessional collaboration (IPC), characterised by shared decision-making and coordinated care, has become a central goal in healthcare education and practice. A key strategy to achieve IPC has been the implementation of interprofessional education (IPE) across curricula, which aims to equip students with collaborative competencies [19]. Through IPE, interprofessional learning (IPL) occurs as students learn with, from and about each other, fostering mutual understanding, respect and teamwork [2].

IPE can be delivered in diverse settings, from classrooms and simulations to clinical environments. A prominent model is the interprofessional training ward (IPTW), where students from multiple professions engage in collaborative clinical practice under supervision while delivering patient care [22, 32]. Unlike traditional placements, IPTWs are deliberately structured to promote shared responsibility and interaction across health professions. Originating in Sweden and now adapted internationally, IPTWs provide a unique opportunity to embed IPL in authentic care contexts where students develop interprofessional competencies through experience, reflection, and clinical supervision.

While IPL has predominantly been studied from the student perspective, previous research has increasingly highlighted the central pedagogical role of educators in facilitating interprofessional learning [18, 27]. Studies of these educators describe facilitation as a complex and demanding practice requiring structured preparation and ongoing support [8, 11]. In IPTWs, this pedagogical role is largely assumed by clinical supervisors placing substantial demands on their pedagogical and organisational capacity [15, 20]. The prerequisites for this role are often underdeveloped, leaving supervisors to navigate interprofessional supervision (IPS) with varying levels of preparation, support and clarity [13, 23].

Within this clinical context, IPS is increasingly recognised as a key enabler of IPL, although its definitions remain inconsistent. Traditionally, clinical supervision has been understood as structured guidance by a senior practitioner within the same profession [6, 16]. More recent work has extended this concept to include supervision across professional boundaries [15, 28]. In this study, interprofessional supervision encompasses supervision across professional boundaries, of interprofessional student teams, and by supervisors from different professions working collaboratively.

Empirical evidence on how supervisors enact IPS in clinical practice remains limited. Existing studies show that supervisors often feel underprepared for interprofessional aspects of their role [10], relying on experience rather than formal training. Previous research has identified competencies such as adaptability and communication as important [7], yet offers little insight into how these competencies are enacted in everyday supervisory practice. Most studies focus on allied health or single-professional perspectives, with limited depth on day-to-day supervisory processes [5, 11, 15, 29].

In summary, although IPL has been widely studied, little is known about how IPS is enacted and experienced by supervisors. This gap limits our understanding of how interprofessional learning is sustained in clinical education.

### Theoretical perspectives

To better understand how IPS is enacted in practice, we draw on two complementary theoretical perspectives: Reeves and colleagues’ conceptualisation of interprofessional work as negotiated practice [25, 26] and Akkerman and Bakker’s [1] theory of boundary crossing.

Reeves and colleagues conceptualise interprofessional work as a negotiated practice, emphasising how collaboration is shaped by professional hierarchies, institutional structures, and power dynamics [25, 26]. From this perspective, collaboration is continuously negotiated as professionals coordinate responsibilities, manage differences in expertise, and work across professional boundaries.

Building on Wenger’s [31] concept of communities of practice, Akkerman and Bakker [1] conceptualise boundary crossing as encounters across sociocultural differences that can generate both tensions and opportunities for learning. They describe how learning emerges when individuals connect practices, negotiate shared meanings, and translate knowledge across professional boundaries.

Together, these perspectives frame IPS as a socially negotiated practice enacted across professional boundaries. They provide complementary lenses for understanding how supervisors negotiate roles and responsibilities, navigate professional hierarchies, and create opportunities for interprofessional learning.

### Aim and research questions

This study aims to better understand how supervisors from different professional backgrounds make sense of and enact interprofessional supervision within interprofessional training wards.

The study addresses the following research questions:

1. How do supervisors from different health professions conceptualise their roles and responsibilities in interprofessional supervision?
2. How is interprofessional supervision practised in interprofessional training wards, and what challenges and opportunities do supervisors perceive?

## Methodology

### Research design

This study employed a qualitative research design using semi-structured interviews to explore experiences of IPS at four IPTWs. Purposeful sampling ensured diverse professional representation. Data were analysed using reflexive thematic analysis [4], an approach that acknowledges the active interpretive role of the researcher in generating themes from the data.

### Study context

This study was conducted at four university hospitals in Sweden: two in Stockholm, one in Norrköping, and one in Linköping. Three of the IPTWs were orthopaedic units and one was a surgical unit. During the study period, students from four healthcare professions collaborated in interprofessional teams, working for two weeks to care for patients undergoing planned orthopaedic or abdominal surgery. Each student team consisted of nursing students, physiotherapy students, medical students, and occupational therapy students.

The students were from two different medical universities, the Karolinska Institutet and Linköping University. The nursing, physiotherapy and occupational therapy students were all in the sixth and final semesters of their undergraduate education. The medical students from Stockholm were in the seventh semester of their eleven-semester programme, and those from Linköping and Norrköping were in their ninth semester. During the two-week placement, students worked in stable interprofessional teams with shared responsibility for patient care. The placement began with an introductory day focusing on team formation and the interprofessional learning objectives, which were common to all professions. Students shared workspaces and participated in joint activities throughout the placement, including daily team meetings, ward rounds and end-of-day reflections, while simultaneously engaging in profession-specific learning and supervision.

Each student at the IPTW had a profession-specific supervisor. However, supervisors’ physical presence varied considerably. Reflecting the routine organisation of clinical work, nursing supervisors were consistently present at the IPTW. Physiotherapy and occupational therapy supervisors were present approximately 50% of the time, as they also covered other wards. Medical supervisors participated as part of their broader clinical responsibilities, which also included surgery and outpatient clinics. None of the supervisors, except the nursing supervisors, were present during evening shifts.

### Participants

Purposeful sampling was used to recruit participants from four professional groups: nurses, occupational therapists, physiotherapists, and physicians. Participants were recruited through professional networks and healthcare institutions, with efforts made to achieve variation in years of professional experience and work settings. The sample was purposefully structured to include equal representation from the four professional groups and variation in both professional experience and experience of supervising in IPTWs. A total of 16 supervisors (four from each profession) participated in the study. Participants’ professional experience ranged from 1 to 35 years, and their experience of supervising at the IPTW ranged from 1 week to 15 years. The adequacy of the sample was assessed using Malterud et al.’s [12] concept of information power. The purposeful sampling strategy, participants’ direct experience of interprofessional supervision, the use of established theoretical perspectives, and the richness of the interview data all contributed to sufficient information power.

### Data collection

Data were collected by the first author (LZ) during the spring and autumn of 2024, through individual semi-structured interviews. Interviews were conducted either in person at participants’ workplaces (*n* = 6) or via Zoom (Zoom Video Communications, Inc., San Jose, CA, USA; *n* = 10). To facilitate detailed descriptions of everyday supervisory practice and make tacit aspects of supervision explicit, interviews began with a prompt inspired by the Interview to the Double (ITTD) technique [14]. ITTD is designed to access tacit knowledge by asking participants to imagine that a double must take over their role and to provide detailed instructions enabling the double to perform convincingly without being recognised as an imposter [17]. The interviews then continued using a flexible semi-structured interview guide exploring participants’ experiences, perceptions and reflections on interprofessional supervision at the IPTWs (supplement). Follow-up questions were used to encourage elaboration and depth. Interviews lasted approximately 45–60 min, were audio-recorded and transcribed verbatim. Participants received written study information and a consent form by email a few days before the interview. Written informed consent was obtained before all interviews.

### Data analysis

We analysed the data using reflexive thematic analysis, following the methodological principles described by Braun and Clarke [4]. In our analysis, familiarisation began during transcription, which was undertaken by the first author (LZ), and continued through repeated reading of the transcripts. LZ manually coded the complete dataset and developed initial codes capturing meaningful features of the data. Initial codes were organised and iteratively regrouped using paper excerpts and analytical notes rather than qualitative software, allowing themes to develop through repeated engagement with the data. As interviews from different professions were conducted and transcribed over time, familiarisation and coding progressed alongside the data collection. As the dataset expanded, patterns and variations across professional groups became increasingly apparent, prompting a more explicit comparative reading of the transcripts. In the later stages of analysis, transcripts were revisited profession by profession to examine similarities and differences across the four professions. Preliminary themes were discussed with the third author (AS) and refined through repeated reference to the original transcripts before the final themes were agreed upon by all authors (LZ, AS, KBL).

As themes were developed, the concepts of negotiated practice [25] and boundary crossing [1] were used as sensitising concepts to further interrogate the developing themes. Alongside the researchers’ disciplinary and educational backgrounds, these frameworks directed attention to how supervisors negotiated their practices in relation to organisational conditions and professional roles, and to how professional boundaries both enabled and constrained interprofessional supervision.

### Reflexivity

The first author (LZ) is a registered nurse with extensive experience of profession-specific and interprofessional supervision, including supervision on several IPTWs. This background provided an in-depth understanding of the clinical and pedagogical context, facilitating sensitivity to nuances in supervisory practice while also shaping the interpretation of the data. To broaden the interpretive scope, the analysis was continuously discussed with the third author (AS), a surgeon, educational leader and clinical education researcher with responsibility for IPTW development. His professional and educational perspective contributed to refinement of the developing themes. The second author (KBL), a professor of higher education pedagogy with no clinical involvement in IPTW, provided an external perspective that challenged assumptions and added analytical depth. Throughout the study, the research team engaged in regular reflexive discussions, critically examining how their respective positions and experiences informed the interpretation of the data.

### Ethical considerations

Ethical approval was obtained from the Swedish Ethical Review Authority (reference no. 2023-07098-01). The study was conducted in accordance with the principles of the Declaration of Helsinki [30]. All participants provided written informed consent prior to data collection and were informed that participation was voluntary, that they could withdraw at any time without consequences, and that confidentiality would be maintained. Data were anonymised and stored securely.

## Results

Sixteen supervisors from nursing, medicine, physiotherapy and occupational therapy participated in the study (Table 1).

**Table 1:** Characteristics of the participating supervisors

Participant	Gender	Years in profession	Years supervising in IPTW	Supervisor training
Nurse 1	F	13	8	Yes
Nurse 2	F	24	5	Yes
Nurse 3	F	7	4	No
Nurse 4	M	5	3.5	Yes
Occupational therapist 1	F	7	3	No
Occupational therapist 2	F	5	2.5	No
Occupational therapist 3	F	2	2	Yes
Occupational therapist 4	M	5	2.5	No
Physician 1	M	NR	5	No
Physician 2	F	6	1	No
Physician 3	F	5	1	No
Physician 4	F	NR	1	Yes
Physiotherapist 1	F	11	3	Yes
Physiotherapist 2	F	35	15	No
Physiotherapist 3	M	8	3	No
Physiotherapist 4	F	2.5	2	Yes

*Abbreviations*: *IPTW* (interprofessional training ward), *NR* (Not reported)

The analysis generated three interconnected themes capturing how supervisors experienced and enacted interprofessional supervision: (1) Learning to swim in uncharted waters, describing how supervisors assumed the role with little preparation or shared expectations; (2) Navigating tensions between profession-specific and interprofessional supervision, illustrating the ongoing balancing of profession-specific and collective responsibilities; and (3) “Just call the nurse”, highlighting how responsibility for sustaining interprofessional supervision gradually became concentrated within the nursing profession.

### Theme A. Learning to swim in uncharted waters

Supervisors entered IPS through different pathways, but regardless of how they assumed the role they described becoming interprofessional supervisors largely through experience rather than by formal preparation. For nurses, physiotherapists and occupational therapists, entering IPS was often described as an active choice, driven by an interest in teaching and interprofessional collaboration. One nurse described wanting *“something more than just being on the floor and I thought the team aspect was truly interesting”* (Nurse 3), while a physiotherapist had returned to the hospital specifically to work on the interprofessional ward, calling it *“a deliberate choice”* (Physiotherapist 3) because she genuinely enjoyed the role. For these supervisors, IPS represented an opportunity for pedagogical engagement and continuous learning: *“When you’re passionate about something*,* you read up on it*,* you want to know more”* (Nurse 3).

Physicians, in contrast, consistently described entering IPS through organisational assignment rather than personal choice. Several portrayed supervision as *“mandatory”* (Physician 1) during residency or as something they first find out about when *“the schedule comes out”* with *“no planned introduction”* (Physician 3). Thus, supervisors entered IPS under markedly different conditions, potentially shaping both their expectations and opportunities to develop in the role.

Regardless of how they entered IPS, supervisors across professions described learning the role with remarkably little formal preparation. One physician recalled that *“I didn’t get any training… it was more like—you’re here*,* so you supervise”* (Physician 3). Even those who had completed generic supervisor training felt that it provided little guidance for supervising across professional boundaries, and several participants expressed a wish for more structured preparation, including opportunities to “*meet or practice together as supervisors”* (Physician 1) before taking on the role. In the absence of such preparation, supervisors relied heavily on informal learning through observing experienced colleagues, exchanging practical advice and gradually developing their own supervisory approaches. As one physiotherapist explained, she had *“picked up a lot”* (Physiotherapist 1) from experienced colleagues.

Supervisors’ reliance on informal learning reflected not only limited pedagogical preparation but also organisational conditions that offered few opportunities to develop as interprofessional supervisors. Fragmented scheduling, competing clinical responsibilities and limited opportunities for joint reflection constrained continued learning and made it difficult to establish confidence and continuity in the role. As one nurse explained, *“I was just pushed in… People think you can just jump in and supervise. However*,* it’s not like that”* (Nurse 1). Consequently, learning to supervise became largely dependent on local colleagues and individual initiative rather than a structured organisational process.

Taken together, these accounts suggest that becoming an IPS supervisor was less a structured transition than an ongoing process of adaptation. Rather than entering an established pedagogical practice, supervisors gradually constructed their supervisory role through experience, peer learning and personal commitment, with formal preparation and organisational support playing only a limited role.

### Theme B. Navigating tensions between profession-specific and interprofessional supervision

Having entered the role, supervisors described interprofessional supervision as a practice that was neither clearly defined nor consistently understood. Rather than replacing profession-specific supervision, IPS required supervisors to navigate responsibilities that often extended across professional boundaries.

Many supervisors found it difficult to articulate what interprofessional supervision actually meant. When asked to define the concept, several supervisors responded with hesitation or humour: *“Oh. No idea.”* (Physician 3), *“Oh*,* that’s a good question”* (Occupational therapist 4) or *“I honestly don’t know what to say”* (Physiotherapist 2) – suggesting that, despite working within an interprofessional training ward, supervisors often lacked a shared conceptual and pedagogical understanding of IPS and, consequently, of their role as interprofessional supervisors.

In the absence of this shared understanding, supervisors also described the supervisory role in markedly different ways. Some primarily understood their responsibility as supporting students from their own profession. As one physician explained, “*You supervise your own profession… but I guess the idea is they’re supposed to learn from each other”* (Physician 2). Others, particularly nurses, described a broader supervisory role extending across professions: *“I’m here for all the professions”* (Nurse 1).

These different understandings became particularly visible when supervisors encountered students from professions other than their own. Many described uncertainty about how far their supervisory responsibility extended. As one occupational therapist explained, *“If they have questions about medicine*,* I can’t truly help. I usually refer them to a physician or a nurse”* (Occupational therapist 2). Nurses, by contrast, often described simultaneously supervising nursing students while informally coordinating the learning of the wider student team, illustrating how different understandings of the supervisory role resulted in different enactments of IPS.

Rather than resolving these tensions, many supervisors learned to work with them. Some deliberately encouraged students to rely on one another instead of immediately providing answers themselves. As one physiotherapist reflected, *“Your job is to help the students interact with each other… sometimes that means sitting on your hands and not jumping in with answers”* (Physiotherapist 2). Similarly, an occupational therapist described using students’ different professional perspectives during feedback by asking, “*What do you think your PT colleague did well?”* (Occupational therapist 4). Several supervisors also emphasised that modelling professional interdependence was itself an important learning objective. As one nurse explained:

*“There’s a risk that supervisors feel they have to take on too many roles—to have all the answers. However*,* I think it’s important to show students that we can’t know everything—and that we always depend on others*,* including other professions”* (Nurse 4).

These negotiations became particularly visible when supervisors assessed students’ learning. Assessment was widely described as one of the most uncertain aspects of IPS because participants lacked shared criteria and often had limited insight into what students should learn. One physician captured this uncertainty succinctly: *“You don’t truly know what the others are doing*,* so how are you supposed to give feedback?”* (Physician 2). Without common assessment frameworks, supervisors frequently relied on profession-specific tools. A physiotherapist explained, *“I just use our regular form. It’s not truly adapted*,* but at least it’s something” (Physiotherapist 1).* Others limited their feedback to aspects they felt qualified to judge. As one occupational therapist noted, *“I give feedback on how they act in the team*,* but not on things I don’t understand. That’s for their own supervisor”* (Occupational therapist 4).

At the same time, several supervisors described developing collaborative approaches that extended beyond profession-specific assessment. They consulted colleagues before evaluations, conducted joint feedback sessions or invited students to reflect together across professional boundaries. One physiotherapist described consulting colleagues before providing feedback by asking, *“What did you see?”* (Physiotherapist 2). Another supervisor explained: *“We started doing joint feedback. It made an enormous difference for the students—and for us”* (Nurse 2).

Taken together, these accounts suggest that interprofessional supervision was characterised less by clearly defined responsibilities than by ongoing negotiation of what the supervisory role itself entailed. In the absence of a shared conceptual and pedagogical understanding of IPS, supervisors developed different understandings of their role, resulting in different ways of enacting interprofessional supervision across professional boundaries. These differences became particularly visible during assessment, where uncertainty about how to evaluate interprofessional learning reflected broader uncertainty about the purpose, scope and pedagogical foundations of IPS.

### Theme C. “Just call the nurse”: The unchosen responsibility and unbalanced leadership of nurses

Across interviews, nurses occupied a distinctive position within interprofessional supervision. While supervisors from all professions contributed to students’ learning, nurses’ continuous presence on the ward and broad overview of patient care meant that they often became the natural point of contact for both students and fellow supervisors. Supervisors across professions described turning to the nurse supervisor whenever questions arose or coordination was needed. As one occupational therapist explained, “*Then*,* I usually talk to the nurse supervisor and say*,* you need to have a conversation with that student”* (Occupational therapist 2).

Nurses themselves described this broader responsibility as both natural and unavoidable. One supervisor reflected: *“We’re supposed to zoom out more*,* see the team as a whole in a way the others don’t. […] We’re always out there among the team”* (Nurse 1). Rather than being formally assigned, this coordinating role appeared to develop through everyday practice. Because nurses remained continuously available while other supervisors moved between different clinical responsibilities, they became the pedagogical infrastructure that allowed IPS to function. For many nurses, this coordinating role also aligned with a strong interest in teaching and interprofessional collaboration, reinforcing their engagement in supervising the student team. As one nurse reflected, *“Doctors don’t truly have that overview… they’re not around the whole day like the rest of us are” (Nurse 4)*. Rather than reflecting differences in motivation or engagement, participants consistently described how different clinical work patterns created unequal opportunities to participate in the everyday supervision of the student team.

This central position for nurse supervisors, however, also carried a largely invisible workload. Several nurses described constantly monitoring students while simultaneously carrying out ordinary clinical work, often supervising without appearing to supervise. One nurse explained: *“I usually follow the students around pretending to tidy up the rooms*,* just so I can make sure it’s safe for the patients”* (Nurse 3). Similarly, supervisors from other professions acknowledged relying on nurses’ ongoing presence. A physician admitted: *“I don’t have time to just sit with them and hang out […] My nursing colleagues usually tell me if something seems off”* (Physician 2). These accounts suggest that much of the pedagogical work sustaining IPS occurred quietly in the background. Rather than protected educational activities, supervision became intertwined with routine clinical work, requiring continuous multitasking and informal coordination. Although nurses occupied this central coordinating position, their leadership was neither straightforward nor uncontested. Supervisors reported situations where professional boundaries complicated their ability to intervene or where their authority depended largely on personal experience rather than formal responsibility. One physiotherapist described “situations where treatment plans were incorrect but intervention was not always straightforward” (Physiotherapist 3). Nurses themselves also emphasised that confidence in the role depended heavily on experience: *“It’s a big difference working with someone like me who’s been here for eight years […] we also have a few who maybe aren’t suited to be nurse supervisors”* (Nurse 1).

Supervisors from other professions likewise described organisational constraints that limited their ability to contribute equally to interprofessional supervision. One occupational therapist explained: *“I had no colleagues… I had to run between places*,* so I wasn’t even 100% on the ward where the students were”* (Occupational Therapist 2).

These accounts suggest that the uneven distribution of supervisory responsibility reflected not only differences between professions but also differences in the organisational conditions under which supervisors worked.

Taken together, these accounts suggest that responsibility for sustaining interprofessional supervision became concentrated within the nursing profession, not because this role was formally assigned, but because organisational arrangements consistently created greater opportunities for nurses to coordinate the interprofessional learning environment. Consequently, leadership of IPS emerged primarily through everyday participation in clinical work rather than through formally designated authority.

## Discussion

This study explored how supervisors from four professions made sense of and enacted interprofessional supervision in interprofessional training wards. Supervisors portrayed IPS as a fragile and demanding practice. Across professions, they described learning IPS largely through experience rather than through structured preparation. They navigated constant tensions between profession-specific and interprofessional goals, while nurses frequently assumed a central coordinating role in supporting student teams. Taken together, these findings suggest that IPS emerged as a distinct pedagogical practice that was continuously negotiated across professional, pedagogical and organisational boundaries.

### Negotiating interprofessional supervision in clinical practice

Supervisors described entering IPS with little formal preparation, instead relying on professional experience and informal discussions with colleagues. Their accounts support previous descriptions of interprofessional facilitation as a complex and demanding educational practice, while also highlighting a gap between these demands and the structured preparation and ongoing faculty development recommended in previous studies [5, 8, 10, 11]. Our findings illustrate what happens when supervisors enter interprofessional supervision without a shared pedagogical understanding. Despite working within the same IPTW model, they developed different understandings of the purpose and enactment of IPS, drawing on profession-specific traditions, individual experience and locally negotiated practices. These differing understandings shaped how supervisors approached feedback, assessment and responsibility for student learning, making the pedagogical purpose of IPS itself an object of negotiation rather than a shared point of departure.

Our findings further illuminate the complex and demanding nature of interprofessional supervision in clinical practice. Previous studies of IPE facilitation have primarily described educators in relation to their role in supporting interprofessional learning [8, 11, 29]. In IPTWs, however, supervisors were not solely responsible for facilitating interprofessional learning. They simultaneously remained responsible for profession-specific supervision while responding to patient care and the practical demands of everyday clinical work. Consequently, IPS did not replace profession-specific supervision but became layered upon it, requiring supervisors to move continuously between complementary pedagogical roles.

Within this layered supervisory role, interprofessional supervision was characterised by ongoing negotiation rather than the enactment of a predefined pedagogical model. Supervisors continuously negotiated educational purposes, supervisory responsibilities, assessment, and leadership while balancing interprofessional learning with profession-specific supervision and patient care. Reeves and colleagues conceptualise interprofessional work as negotiated practice, in which roles, responsibilities and professional contributions are shaped through interaction across professional and organisational boundaries [25, 26]. Our findings extend this perspective by showing that such negotiation also characterises the pedagogical domain of interprofessional supervision.

### Boundary crossing as an opportunity and strain

While boundary crossing has long been recognised as fundamental to interprofessional learning [27], our findings illustrate how supervisors facilitated and enacted boundary crossing through their everyday supervisory practices.

Supervisors encouraged students to seek expertise from other professions, engaged in joint feedback processes, discussed assessments across professional boundaries and modelled collaborative practice. Viewed through Akkerman and Bakker’s [1] framework, these practices exemplify processes of coordination and co-construction of meaning across professional boundaries.

Several organisational and pedagogical resources at the IPTW appeared to support these processes. For students, shared learning objectives, structured reflection, common communication practices, and shared responsibility for patient care provided common reference points across professions. Such resources resemble the mediating artefacts and shared language highlighted as important for supporting boundary crossing in health professions education [1, 21].

For supervisors, comparable pedagogical resources were less evident. Joint feedback sessions and collegial discussions sometimes provided shared reference points for coordinating supervision and developing a common understanding of students’ interprofessional learning. Shared approaches to assessing interprofessional learning and a common pedagogical framework were notably absent.

Viewed through the lens of boundary crossing, supervisors’ accounts primarily reflected processes of identification and coordination. Participants became increasingly aware of differences in professional perspectives, responsibilities, and approaches to supervision, while simultaneously developing local ways of coordinating supervision across these differences. The results also show examples of reflection, with supervisors describing learning from one another and reconsidering aspects of their own supervisory practice, although they rarely described their supervisory role or professional identity as fundamentally transformed through interprofessional supervision. The tensions supervisors described should not be interpreted as signs of unsuccessful collaboration. Rather, they were an inherent and expected feature of working across professional boundaries.

However, opportunities and responsibilities for boundary-crossing were not equally distributed across professions. While supervisors across professions described practices that supported collaboration, nurses were uniquely positioned to act as brokers, continuously connecting professional perspectives, coordinating student learning, and sustaining the interprofessional learning environment.

Akkerman and Bakker describe brokerage as both enabling and vulnerable, since brokers facilitate collaboration while simultaneously carrying the burden of maintaining connections across boundaries [1]. Our findings resonate with this perspective by illustrating how brokerage became a central mechanism for sustaining interprofessional supervision.

### Informal leadership and uneven supervisory responsibility

Although nurses’ brokerage was central to sustaining interprofessional supervision in everyday practice, this responsibility remained largely informal and was rarely accompanied by explicit institutional recognition or pedagogical support. Nurses consequently emerged as the informal coordinators of IPS, becoming the natural point of contact for both students and supervisors when challenges arose. This role appeared to reflect a combination of organisational conditions, continuous presence in the ward and strong engagement in interprofessional learning rather than formal authority.

While this position provided continuity and coherence for both student learning and interprofessional collaboration, it also concentrated pedagogical and relational responsibility within one profession. Reeves and Hean, [24] use the notion of structural asymmetries to describe how organisational and professional structures can create unequal conditions across professions. Viewed through this perspective, our findings suggest that opportunities to shape and sustain IPS were not evenly distributed across professions. Rather than being embedded within formal supervisory structures, leadership and brokerage emerged through everyday clinical practice.

### Implications for practice

Previous work has emphasised the importance of preparing facilitators for interprofessional learning [8, 11]. Our findings extend this argument by suggesting that preparation should foster a shared pedagogical understanding of IPS as an interprofessional supervisory practice embedded in clinical work. Such preparation should help supervisors negotiate the relationship between profession-specific and interprofessional learning, facilitate productive boundary crossing, assess collaborative competence and develop shared responsibility for student learning. A shared understanding of the purpose of IPS may also help supervisors recognise interprofessional supervision as a collective responsibility rather than an additional task attached to individual professional roles.

Beyond implications for supervisor preparation, our findings also raise questions about how clinical workplaces support and sustain IPS. Responsibility for sustaining interprofessional supervision was unevenly distributed across professions, reflecting differences in professional roles and in opportunities to participate in supervision. A possible response would be to reshape clinical work within IPTWs. However, Billett’s conceptualisation of workplaces as learning environments cautions against applying assumptions derived from educational institutions uncritically to workplace learning [3]. Clinical workplaces have their own goals, practices and structures of participation, which shape the opportunities available for learning. We therefore question whether equal participation is a realistic expectation when interprofessional supervision is embedded within everyday clinical work. From this perspective, differences in participation reflect how professional work is enacted in clinical practice rather than organisational shortcomings. The organisational challenge is therefore less to equalise participation than to recognise, coordinate and support the different forms of participation that clinical work affords within a shared pedagogical approach to IPS.

Although IPTWs provide a distinctive educational setting, the challenges identified in this study are likely to arise wherever interprofessional supervision is embedded in authentic clinical practice. Supporting IPS therefore requires attention not only to supervisor preparation but also to the workplace conditions that enable interprofessional supervision to develop and be sustained in everyday clinical practice.

### Methodological reflections

The inclusion of supervisors from nursing, medicine, occupational therapy and physiotherapy across four IPTWs provided variation in professional background, supervisory experience and organisational context, enabling a rich exploration of how interprofessional supervision was understood and enacted across different settings. Participants also represented different stages of experience with IPS, from newly appointed supervisors to those with several years of involvement, allowing the analysis to capture perspectives across different stages of supervisory development. As the study was conducted within Swedish IPTWs, the transferability of the findings to other educational and healthcare systems should be considered with caution.

The interviews were conducted by a nurse researcher, whose professional background may have facilitated rapport while also shaping the interaction and the accounts generated through shared professional understandings. However, analysis was undertaken within a multidisciplinary research team with complementary clinical and educational expertise, allowing interpretations to be discussed, challenged and refined throughout the analytic process. Consistent with reflexive thematic analysis, the findings should be understood as interpretive and co-constructed rather than objective representations of participants’ experiences.

Within these contextual and methodological boundaries, the study provides a nuanced understanding of how interprofessional supervision is understood and enacted in authentic clinical practice.

## Conclusion

Interprofessional supervision should be understood not simply as a method for facilitating interprofessional learning, but as a distinct pedagogical practice embedded within everyday clinical work. Supervisors need opportunities to develop a shared pedagogical understanding of IPS and to prepare for the ongoing negotiations involved in balancing profession-specific and interprofessional learning. At the same time, organisations need to recognise that opportunities for supervision are shaped by different forms of professional participation. Strengthening IPS therefore depends not only on preparing individual supervisors but also on creating pedagogical and organisational conditions that recognise, coordinate and support these complementary contributions across professional boundaries.

## Supplementary Information

Supplementary Material 1.

## Data Availability

The datasets generated and analyzed during the current study are not publicly available due to confidentiality but are available from the corresponding author on reasonable request.

## Abbreviations

IPE: Interprofessional education
IPL: Interprofessional learning
IPS: Interprofessional supervision
IPTW: Interprofessional training ward
WHO: World Health Organization
