# Supplementary material for: Supervising across professions: insights into the art of interprofessional clinical supervision
Source: BMC Med Educ. 2026 Sep 9;26:1450. doi: 10.1186/s12909-026-10355-y (PMC13560351; doi:10.1186/s12909-026-10355-y)
Supplement: Supplementary file 1 [file 12909_2026_10355_MOESM1_ESM.docx]

Interview guide – Supervising across profession

**Research questions:**

1. How do supervisors from different health professions conceptualize their roles and responsibilities in interprofessional supervision?
2. How is interprofessional supervision practiced in the context of an IPTW, and what challenges and opportunities do supervisors perceive?

**Overview questions / background**

1. What profession do you belong to?
2. Which professions are part of your supervisor team?
3. How long have you worked as a (profession)?
4. How long have you been working as an Interprofessional Supervisor?
5. How did you end up in this Interprofessional Training Ward?
6. Have you taken a supervisor course? If yes, which one?

**Prompt “Interview to the double” (ITTD)**

I would like you to start the interview by imagining that you are going to introduce a new colleague in interprofessional supervision who will be able to act as a duplicate of yourself. You do this so that you are on leave for six months. You want to travel away to a place and refuel with energy, but the boss doesn't want to give you a leave of absence. Your duplicate will take over your work the next day and you will not be able to contact the duplicate from where you are traveling. You need to provide your duplicate with the necessary detailed instructions to ensure that you and your duplicate are not exposed. Prepare your duplicate for any difficulties that may arise in the interprofessional supervision? How would that sound?

**Questions related to organisation/collaboration in the supervisor team**

- How do you set up the interprofessional supervision?
- How do you feel when the other supervisors in the supervisor group supervise your profession?

**Questions related to supervision techniques**

- What is supervision for you?
- What is interprofessional supervision for you?
- Is there anything in particular in your education or previous experiences that you think makes it easier when supervising interprofessional student groups and specifically when supervising another profession?
- Is there anything you find difficult about supervising groups with students from different professions? If yes, what?
- If you think specifically about the supervision of students from other professions, how do you feel it is to supervise these?
- Does it matter what profession they belong to? If yes, why do you think this is so?
- How do you feel that it affects learning that the supervision is also interprofessional?
- How do you feel about assessing students' knowledge and abilities? What makes it easy/difficult?

**Questions about the emotional experience of the supervisor assignment**

- Can you tell me about a positive experience you have had when supervising students from other professions? What made it positive? What was your role in the situation? What did you think of? Was there anything the student did that contributed to the positive experience?
- Have conflicts ever arisen when supervising students from other professions?
- Have you experienced a situation at work where you were unsure about the supervision while having a student with you?
- Have you been a supervisor for students that you have worried about, about their suitability? Have you experienced that a student has done something inappropriate? How was it for you? Have/would you have handled it differently if the student was from another profession?
